# Engineered high endurance in WO_3_-based resistive switching devices via a guided filament approach

**DOI:** 10.1126/sciadv.adt9789

**Published:** 2025-05-16

**Authors:** Ziyi Yuan, Babak Bakhit, Yi-Xuan Liu, Zhuotong Sun, Giulio I. Lampronti, Xinjuan Li, Simon M. Fairclough, Benson K. Tsai, Abhijeet Choudhury, Caterina Ducati, Haiyan Wang, Markus Hellenbrand, Judith L. MacManus-Driscoll

**Affiliations:** ^1^ Department of Materials Science & Metallurgy, University of Cambridge, 27 Charles Babbage Rd, Cambridge CB3 0FS, UK.; ^2^ Electrical Engineering Division, University of Cambridge, JJ Thomson Avenue, Cambridge CB3 0FA, UK.; ^3^ Thin Film Physics Division, Department of Physics (IFM), Linköping University, Linköping SE-58183, Sweden.; ^4^ School of Materials Engineering, Neil Armstrong Hall of Engineering, Purdue University, 701 West Stadium Avenue, West Lafayette, IN 47907-2045, USA.

## Abstract

Resistive switching devices are promising candidates for the next generation of nonvolatile memory and neuromorphic computing applications. Despite the advantages in retention and on/off ratio, filamentary-based memristors still suffer from challenges, particularly endurance (flash being a benchmark system showing 10^4^ to 10^6^ cycles) and uniformity. Here, we use WO_3_ as a complementary metal-oxide semiconductor–compatible switching oxide and demonstrate a proof-of-concept materials design approach to enhance endurance and device-to-device uniformity in WO_3_-based memristive devices while preserving other performance metrics. These devices show stable resistive switching behavior with >10^6^ cycles, >10^5^-second retention, >10 on/off ratio, and good device-to-device uniformity, without using current compliance. All these metrics are achieved using a one-step pulsed laser deposition process to create self-assembled nanocomposite thin films that have regular guided filaments of ≈100-nanometer pitch, preformed between WO_3_ grains and interspersed smaller Ce_2_O_3_ grains.

## Teaser

A materials design greatly improves the endurance of WO_3_ memory devices, advancing future computing technologies.

## INTRODUCTION

The exponential increase in global data generation—reaching 79 zettabytes in 2020 and continuously growing (*1*)—has intensified the demand for more efficient data storage and processing technologies. Meanwhile, the exponential growth of information processing demands from artificial intelligence requires a revolution in the current von Neumann architecture—the traditional computing model separating memory and processing units—and therefore, neuromorphic computing, which aims to mimic the neural activity of the human brain for advanced artificial intelligence applications, catches attention (*2*). Resistive switching devices have emerged as promising candidates to meet these challenges. The basic principle of resistive switching involves changes in resistance states upon the application of electric fields. Metal oxides, conductive organic molecules, and other hybrid materials are common choices for the switching medium (*3*, *4*). Metal oxides such as TiO_2_ (*5*), HfO_2_ (*6*, *7*), TaO*_x_* (*8*), SiO_2_ (*9*), and WO*_x_* (*10*, *11*) have been extensively studied. Although excellent endurance performance of up to 10^12^ cycles has been reported for example in TaO*_x_* (*12*), limited numbers of devices and sometimes noisy data suggest that such demonstrations are exceptional and fundamental endurance problems for filamentary resistive switching persist. WO*_x_* has garnered large attention owing to its simple complementary metal-oxide semiconductor (CMOS) compatibility (*13*), low cost (*14*), and multiple oxidation states (*15*), wherein the intrinsic oxygen vacancies can play an important role in the filamentary resistive switching mechanism (*16*).

Despite many advances in performance enhancement and fundamental understanding, most filamentary systems undergo random formation and rupture of conducting filaments. The unpredictability of these paths can result in problems such as high forming voltage (typically larger than the voltage required to switch the devices) (*17*), high device-to-device and cycle-to-cycle variation, and low endurance (*18*), except for rare examples as mentioned before. The variability not only affects the performance and endurance of individual devices but also poses challenges for large-scale integration and manufacturing consistency (*2*, *19*). These difficulties in general oxide switching materials are also challenges in WO*_x_*-based devices, where the variability in filament formation within the WO*_x_* layers causes inconsistent switching behavior, affecting the overall endurance of the devices. Although WO*_x_*-based memory devices with interfacial switching may have better uniformity, they lack sufficiently high retention (*20*–*22*).

To address the challenges of filamentary systems, researchers have explored a variety of strategies to enhance the stability of the filament formation. For example, Si doping in WO*_x_* can localize filament conduction paths and improve endurance (*23*). In addition, engineered grain boundaries can provide preferential regions for filament formation in polycrystalline HfO_2_ (*24*). Furthermore, embedding Ag nanoparticles in SiO_2_-based devices can aid filament formation (*25*). Vertically aligned nanocomposites (VANs) also prove controlled filament formation in both interface- (*26*) and filament-dominated memristive devices (*27*–*30*), where, additionally, reduced switching voltages were observed. Nevertheless, the properties of endurance, retention, and uniformity are still unbalanced and far from satisfactory for industrial-scale applications.

Here, we demonstrate a thin film nanocomposite filamentary approach to achieve strong improvement in the combined performance metrics. We present a previously unreported class of WO_3_-based resistive switching devices by combining the advantages of WO_3_ (with high concentration of intrinsic oxygen vacancies) and Ce_2_O_3_ (with charge-carrier blocking structure) (*31*, *32*) to construct a nanocomposite that guides the formation of filaments. This nanocomposite filamentary design was validated in nanocomposite thin films, deposited in a single-step pulsed laser deposition (PLD), with a 1:1 cation ratio of WO_3_ and Ce_2_O_3_ that leverage a unique phase-separated structure. Structure characterization revealed that the films consist of WO_3_ grains interspersed with Ce_2_O_3_ grains, promoting confined guided filaments of 5 to 10 nm width and ≈100-nm pitch at grain interfaces between WO_3_ and Ce_2_O_3_. Conductive atomic force microscopy (CAFM) measurements provide direct evidence of the guided filament formation at the grain interfaces. As a result, an endurance improvement in WO_3_-based devices of one to two orders of magnitude can be achieved without sacrificing any other performance metric such as retention and uniformity. These findings provide a paradigm for energy-efficient memristors design with reliable performance.

## RESULTS

### Electrical performance

**Fig. 1.**
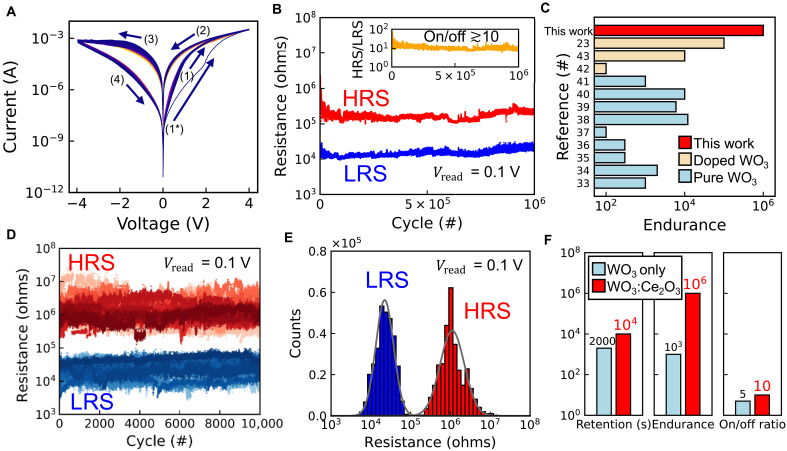
Performance of the WO_3_:Ce_2_O_3_ nanocomposite devices of this study. (**A**) Initial 1000 *I*-*V* curves of a device on the nanocomposite film. The top electrode is Pt with a diameter of 100 μm. A soft forming process (1*) is observed during the first voltage application but does not require a higher maximum voltage than subsequent switching cycles. The switching sequence is indicated by numbered arrows (1 to 4), and the 1000 curves overlap each other closely. (**B**) Pulsed endurance measurement showing that the LRS and HRS are maintained for at least 10^6^ switching cycles. The set and reset voltage were ±4 V with a read voltage of 0.1 V. Inset: On/off ratio across the 10^6^ switching cycles. (**C**) Endurance comparison with state-of-the-art single-layer filamentary resistive switching devices of doped or pure WO_3_ as the functional layer. (**D**) Cycling endurance of 10^4^ switching cycles from 30 different devices. Different transparency in color indicates different devices. Set, reset, and read voltages were 4, −4, and 0.1 V, respectively. The resistance values remain stable, indicating reliable switching performance. (**E**) Resistance distributions of the LRS and HRS values in (D). The tails of the fitted normal distributions do not overlap, indicating a stable common memory window. (**F**) On/off ratio, endurance, and retention compared with reference device of the pure WO_3_ film, which were prepared by identical sample fabrication methods and electrode materials. A large improvement in endurance is achieved in the nanocomposite devices.

Our WO_3_:Ce_2_O_3_ nanocomposite thin films demonstrate exceptional resistive switching performance. The initial 1000 current-voltage (*I*-*V*) curves of a device based on the optimized nanocomposite are shown in Fig. 1A, demonstrating a clear hysteresis. A gradual forming process is observed during the first voltage increase (1*), but it does not require a larger maximum voltage than the subsequent switching process (4 V for forming and switching) nor a current compliance. The optimization of the film deposition is discussed later. For the measured devices in Fig. 1, the top electrodes were sputtered Pt with a diameter of 100 μm and the common bottom electrode was a (001)-oriented single-crystal 0.5 wt % Nb-doped SrTiO_3_ (Nb:SrTiO_3_) substrate. These devices show outstanding stability over 1000 *I*-*V* cycles with minimal cycle-to-cycle variation. The device endurance, a critical factor for memory applications, is shown in Fig. 1B. After 1 million switching cycles, the device consistently still exhibited two distinct resistive states. Although the high resistance state (HRS) and low resistance state (LRS) exhibited some fluctuations in the first 1000 cycles, the device reached a stable situation with an on/off ratio consistently ≳10. Figure 1C compares the endurance of our devices with other filamentary resistive switching devices that have single-layer pure or doped WO_3_ as the functional layers, including pure WO_3_ studies after 2010 (*33*–*41*), and all three doped WO_3_ studies (*42*, *43*, *23*). Our devices demonstrate exceptional endurance performance compared with other literature reports. Although up to 10^7^ endurance cycles were claimed for a nonfilamentary WO*_x_* system (*44*), those devices suffered from relatively short retention. Here, we demonstrate a remarkable improvement in endurance across filamentary WO_3_-based devices. Figure 1D shows the device-to-device variability of the endurance performance, where 30 devices were measured up to 10^4^ switching cycles. The resistance values among these devices remain stable; the 60 endurance traces show no overlap, and the overall memory window is maintained around 10 for each device. The endurance measurements on multiple devices further emphasizes their uniformity, which is essential for nonvolatile memory applications. The distribution of LRS and HRS values over these devices is shown in Fig. 1E. Thirty devices with 10^4^ cycles yield a total of 3 × 10^5^ HRS and LRS values. The HRS and LRS distributions of the summed points could be fitted with normal distributions centering at 2.2 × 10^4^ and 1.1 × 10^6^ ohms, respectively, without any overlap in the tails of the fitted curves. There is not a single device showing an LRS higher than the lowest possible HRS value among all devices, and vice versa, giving excellent device-to-device uniformity. In addition to the pulsed endurance measurements, we also demonstrated device-to-device uniformity in dc switching, presented in fig. S1. The comparative analysis of pure WO_3_ films is shown in Fig. 1F, with measurement details of pure WO_3_ films provided in fig. S2. The data illustrate the enhancement in endurance of our nanocomposite devices compared with the pure WO_3_ devices fabricated by identical fabrication methods and electrode materials, highlighting the intrinsic advantages of the nanocomposite approach. X-ray photoelectron spectroscopy (XPS) analysis in fig. S3 revealed that the WO_3_ grains in the WO_3_:Ce_2_O_3_ nanocomposite have a higher fraction of oxygen vacancies compared to pure WO_3_. This shows that adding Ce to WO_3_ enhances oxygen vacancy formation in WO_3_, which likely contributes to the improved switching performance.

**Fig. 2.**
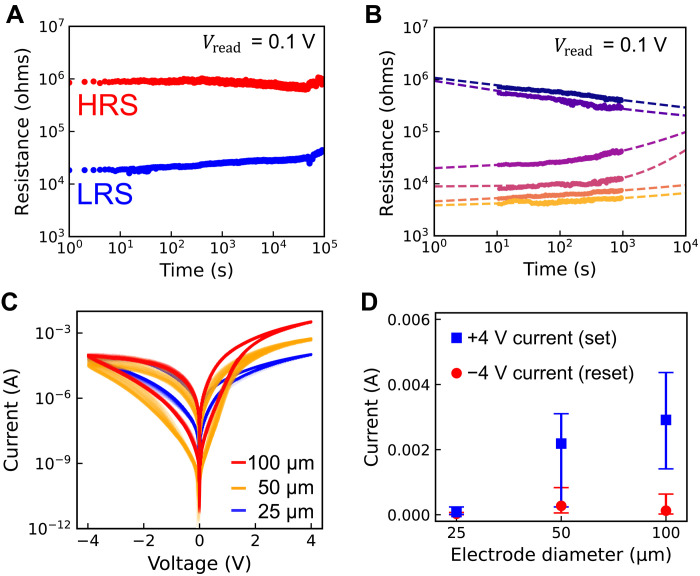
Retention measurement and electrode area dependence of the WO_3_:Ce_2_O_3_ nanocomposite film devices. (**A**) Two-state retention measurements. The set, reset, and read voltages were the same as in Fig. 1B. The LRS and HRS can be clearly distinguished with on/off ratio ≳10 in the measured 10^5^ s. (**B**) Multiple-state retention measurement achieved using five different set voltages. Reset voltage and read voltage were the same as in Fig. 1B. Retention was measured for 10^3^ s. Dashed lines on top are fits to the power law $R=A+B\cdot t^{\beta }$ with A,B>0 and ∣β∣<1. Under the assumption that state decay follows this analytic shape, multistate retention could exceed 10^4^ s. (**C**) Fifty-cycle *I*-*V* curves of three devices with top electrode diameters of 25, 50, and 100 μm. (**D**) Dependence of the currents at +4 and −4 V in the *I*-*V* loops on the electrode size, i.e., the current in the turning point of the *I*-*V* curves, corresponding to the currents at “set” and “reset” operations, respectively. Ten devices each with 25-, 50-, and 100-μm top electrode diameter were tested for 50 cycles. Error bars include the maximum and minimum current of the 50 cycles from each of the 10 devices.

As shown in Fig. 2A, the binary state retention was monitored up to 10^5^ s at room temperature. This outperforms or meets all studies that we compared for endurance in Fig. 1C (see fig. S4 for retention comparison). Despite this leading result, future efforts could improve retention further—e.g., through the addition of a carrier-blocking layer (e.g., Al_2_O_3_) or optimizing the postdeposition annealing temperature, both strategies validated in other works (*45*, *46*). Advancing beyond binary states, Fig. 2B demonstrates the devices’ potential for multilevel memory applications. Through five set voltages, 0.3, 1.5, 2.1, 3.6, and 4, and one reset voltage −4 V, with a read voltage of 0.1 V as for the endurance measurements, we achieved six distinguishable resistance states without requiring any current compliance, which is often a prerequisite for filament-based multilevel devices (*47*). The resistance-time characteristics fit well to a power law $R=A+B\cdot t^{\beta }$ with A,B>0 and ∣β∣<1, adhering to the Curie–von Schweidler law that indicates the time-dependent charge redistribution in dielectrics (*48*). The average *R*^2^ for these fits is greater than 0.9. Extrapolated power law fitting results were displayed as the dashed line on top of the scatter plot. This multistate capability was monitored up to 10^3^ s and could be sustained beyond 10^4^ s if the states continue to follow the analytic expression. To evaluate analog multilevel capabilities, we performed additional pulsed measurements with increasing numbers of fixed voltage pulses and pulses of incremental voltage amplitudes, as shown in fig. S5. Multiple intermediate conductance states can be achieved by the application of continuous pulses, mimicking synaptic weight adjustments in biological systems. Each successive voltage step results in a controlled potentiation or depression. We demonstrated analog switching with two different pulse schemes and with pulse length close to our endurance measurement. Notably, the final conductance achieved in a potentiation cycle depends on the pulse scheme—a fixed-amplitude protocol drove the device into a higher conductance state than an incremental-amplitude protocol with fewer high-voltage pulses. This is because the total electrical stimulus and the device’s state evolution differed between the two schemes. Both measurement protocols confirm that the device can achieve and maintain multiple analog states, making it highly suitable for neuromorphic computing applications that require gradual weight updates and energy-efficient synaptic processing.

From the *I*-*V* curves in Fig. 1A, the devices demonstrated a gradual forming process during the first voltage increase. An important indicator of the underlying switching mechanism is the presence or absence of an area dependence of the device currents. This is investigated in Fig. 2 (C and D). Figure 2C compares the 50 *I*-*V* curves of one 100-μm, one 50-μm, and one 25-μm device. Here, it should be pointed out that, although the 25-μm device switched with ±4 V, different from the larger devices, it required an additional forming step of 6 V to undergo qualitatively the same switching loop. This extra forming step is discussed later. Figure 2D illustrates the current at the switching voltages of 4 V and −4 V, i.e., the current in the turning point of the *I*-*V* curves. The current scales with area at 4 V but not at −4 V. Conventionally, the current in a filament-dominated device shows no area dependency because the current would pass through one filament regardless of the electrode area. The inconsistency of area scaling behavior of set and reset current indicates that the filaments may not be continuous through the entire film. Further analysis of the switching mechanism will be discussed later.

### Structural and compositional analysis

**Fig. 3.**
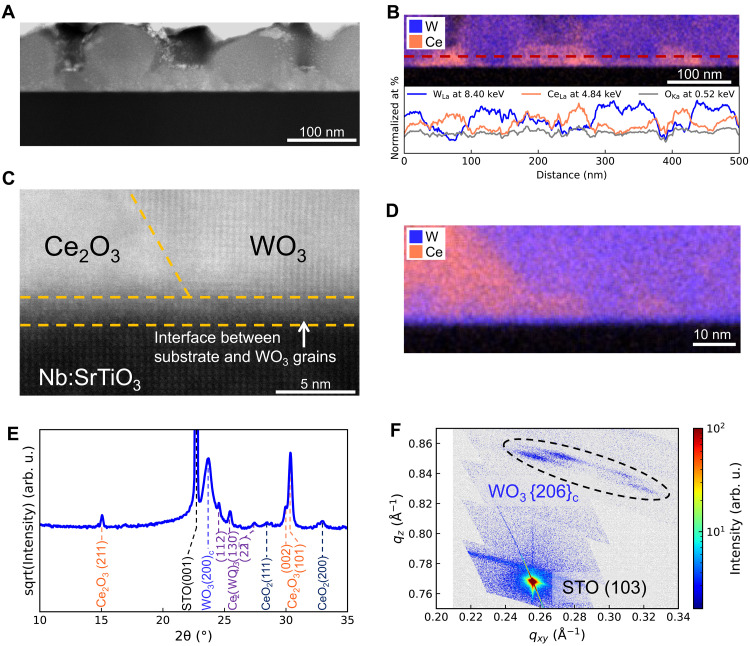
STEM-EDS and XRD analysis of the WO_3_:Ce_2_O_3_ nanocomposite film of this study. (**A**) STEM–HAADF image of the WO_3_:Ce_2_O_3_ nanocomposite thin film deposited on a Nb:SrTiO_3_ substrate. (**B**) EDS map of the image in (A), showing the structure: large spherical W-rich grains segregated by smaller pyramidal Ce-rich grains. In-plane EDS line scans of W, Ce, and O atomic % (at %) were taken across the EDS map, indicated with a red dashed line. (**C**) High-resolution HAADF image at a ternary interface, showing a thin epitaxial layer formed on the substrate prior to the formation of any grains. (**D**) High-resolution EDS map, which clearly illustrates that the thin epitaxial layer is WO_3_. (**E**) XRD 2θ-ω scan of the optimum nanocomposite thin film with all peaks labeled. arb. u., arbitrary units. (**F**) Reciprocal space map near the (103) reflection of SrTiO_3_ showing good lattice alignment of WO_3_ to the substrate.

The nanostructure and phase composition of the nanocomposite film are shown in Fig. 3 (A and B), where high-resolution scanning transmission electron microscopy (STEM)–high-angle annular dark field (HAADF) imaging and energy-dispersive x-ray spectroscopy (EDS) mapping confirm the microscopic structure of the nanocomposite film. Figure 3A reveals spherical, large grains (80 to 100 nm) that grow throughout the film thickness (≈100 nm), interspersed with pyramidal smaller grains (up to 100 nm in diameter), which fill the regions between the larger grains, with ≈30 nm in height. Complementing the STEM-HAADF images, Fig. 3B shows distinct elemental distributions through EDS mapping of W and Ce. The large spherical grains are W-rich, whereas the smaller grains in between are Ce-rich. The EDS line scan was sectioned about 10 nm above the substrate-film interface to reveal the normalized atomic percentage of W, Ce, and O. These scans of W and Ce reveal the alternating presence of W-rich and Ce-rich phases. The O content stays approximately the same across the two different phases. The atomic percent EDS maps of W, Ce, O, Sr, and Ti are presented in fig. S6. All EDS data were analyzed by nonnegative matrix factorization (NMF) (*49*) with details presented in fig. S7. NMF emphasizes the presence of two distinct phases in the nanocomposite: a strong W L_α_ peak in Factor 1 showing the large W-rich grains, and a strong Ce L_α_ peak in Factor 2 showing the small Ce-rich grains in between. This nanostructure is validated by AFM measurements in fig. S8, showing a uniform distribution of spheroidal grains across the film, whose dimensions and spacing align with the observed STEM patterns.

High-resolution HAADF and EDS maps at a ternary interface of a W-rich grain, a Ce-rich grain, and the substrate are shown in Fig. 3 (C and D). As we show later using conductive AFM, conducting filaments form along these interfaces. There is an epitaxial layer of WO_3_ that initially forms on the top of the substrate. The formation of a low-energy epitaxial layer of WO_3_ is consistent with a previous study, which shows that the first layer of the deposited film has low interfacial energy with the substrate (both perovskite structures) to keep the total energy as low as possible (*50*). Then, growth of W-rich grains and the nucleation and growth of Ce-rich grains occur on this interfacial WO_3_ seed layer, an ideal vertically aligned bottom-up structure (*51*). This is likely because the Ce-rich phase lacks a low-energy interface to the W-rich grains and the substrate. We observe from Fig. 3D that some Ce atoms substitute W atoms in the WO_3_ grains. Substitution of W^6+^ with Ce^3+^ within the WO_3_ cation sublattice results in the formation of oxygen vacancies or hole charge carriers to maintain the charge neutrality. The large size mismatch between W^6+^ and Ce^3+^ [Ce^3+^ ionic radius size in sixfold coordination is almost twice as large as W^6+^, ionic sizes ~1.01 Å for Ce^3+^ and ~0.6 Å for W^6+^ (*52*)] induces a large lattice strain that makes the full substitution energetically unfavorable. Thus, this leads to the formation of a Ce-rich phase in between the W-rich grains instead.

Comprehensive x-ray diffraction (XRD) analysis reveals complex crystallographic orientations in the nanocomposite thin films. The 2θ-ω scan of the film, shown Fig. 3E, confirms the coexistence of γ-WO_3_ (*P*21/*n*) and α-Ce_2_O_3_ (*P*$\bar{3}$*m*1) phases. The only phase showing a consistent off-plane orientation is γ-WO_3_ with two similar but not equivalent orientations, (200)_m_ and (020)_m_, in monoclinic notation at ≈23°, denoted as (200)_c_ in cubic notation in the figure. The *c* axis of the monoclinic unit cell matches more closely the lattice constants of the substrate and consequently always lies in plane. Cubic Miller indices are used in this content for clarity because of the strong cubic pseudosymmetry of the unit cell. However, no evidence for an epitaxial relationship was observed in the Ce_2_O_3_ grains, indicated by the presence of multiple Ce_2_O_3_ peaks in the 2θ-ω scans, specifically the (002) and (101) peaks. Such findings underscore the disparate growth behavior of these two phases within the nanocomposite structure. In addition to the main peaks arising from WO_3_ and Ce_2_O_3_, there are low-intensity peaks that have been assigned to the formation of a complex Ce-W intermediate Ce_2_(WO_4_)_3_ phase and a small amount of CeO_2_. Detailed analysis with Rietveld refinement, which confirms the phase identification of the 2θ-ω scans, is shown in fig. S9. In the reciprocal space map, shown in Fig. 3F, four monoclinic WO_3_ reflections are present: (260)_m_, ($\bar{6}0\bar{2}$)_m_, ($\bar{6}$02)_m_, and (06$\bar{2}$)_m_ from left to right, denoted as {206}_c_ in the figure near the (103) reflection of SrTiO_3_, which suggests a robust alignment of the WO_3_ lattice with the substrate. The presence of four rather than six peaks from monoclinic WO_3_ also proves that the *c* axis lies in plane and matches the lattice constant of SrTiO_3_. To optimize the film growth, depositions were performed under a series of temperatures, ranging from 400° to 900°C. Figure S10 compares the 2θ-ω scans of different deposition temperatures, showing the influence of the growth temperature on the crystalline quality of the nanocomposite thin films. Only after 800°C can we observe the peaks of WO_3_ (200)_c_ and Ce_2_O_3_ (101), suggesting a threshold temperature between 700° and 800°C that facilitates crystalline growth. This temperature-dependent behavior emphasizes the importance of sufficiently high thermal kinetics in achieving desired materials nanostructures. Devices with the nanocomposite switching layers deposited at ≥800°C show resistive switching properties. Overall, the devices with the nanocomposite thin films grown at 900°C demonstrate the most stable *I*-*V* curves (see fig. S10). A temperature of 900°C facilitates the growth of a minor ternary Ce_2_(WO_4_)_3_ phase with (112) and (130) orientations, as observed in fig. S11, which is less obvious at temperatures of <900°C. The increased presence of these phases can also potentially enhance the formation of the conducting filaments at much lower forming voltages. The electrical data shown in Figs. 1 and 2 were obtained from the devices with the nanocomposite switching layers deposited at 900°C, referred to as the optimum temperature.

### Observation of guided filament formation

**Fig. 4.**
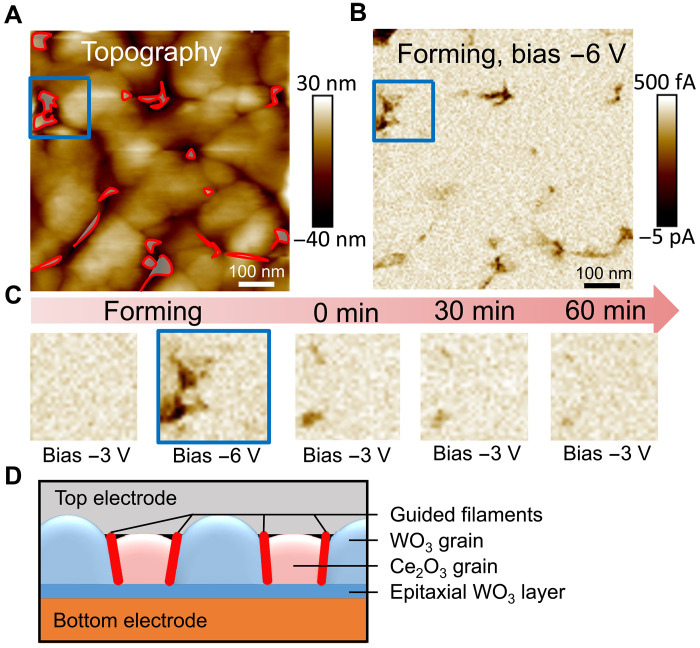
Mechanism explanation of resistive switching in the WO_3_:Ce_2_O_3_ nanocomposite thin films through in situ observation of conducting filaments preferentially formed at the WO_3_/Ce_2_O_3_ vertical interfaces. (**A**) CAFM surface topography. (**B**) CAFM current image acquired immediately after the forming process. The high-current areas (current > 2 pA) are highlighted in their corresponding regions in (A), demonstrating that the filaments are preferentially formed at the vertical interfaces between the WO_3_ and Ce_2_O_3_ phases. (**C**) CAFM images of the marked region in (A) and (B) during different stages of the forming process, with the bias and relative time labeled. The evolution of the images indicates the formation and recovery of conducting filaments. (**D**) Schematic diagram of the design of guided conducting filaments. The filaments are preferentially formed at the vertical interfaces of the nanocomposite film, connecting the top electrode to the epitaxial WO_3_ layer.

Through this nanocomposite architecture, which provides a unique combination of both WO_3_ and Ce_2_O_3_ properties, we carefully engineer preferential sites for the formation of conducting filaments, whose forming voltages are in the same range as the set voltages. An AFM surface topography image in Fig. 4A shows the initial landscape of our measurement region, which corresponds to subsequent CAFM measurements. The discernible bright pillars represent the WO_3_ grains, whereas the surrounding darker regions indicate the smaller Ce_2_O_3_ grains, in agreement with the STEM-HAADF images in Fig. 3A. Figure 4B shows the current map of the nanocomposite film after a forming process of −6 V. Note that the CAFM probe was grounded during the measurement and voltage was applied to the substrate, i.e., the voltage polarity is opposite to that of the *I*-*V* curves. Regions displaying high negative current values indicate the nanoscale regions where the conducting filaments form, and these high-conductivity regions are highlighted in the corresponding areas on the topography in Fig. 4A. These regions appear at the vertical interfaces between WO_3_ and Ce_2_O_3_ grains, which serve as preferential sites for conducting filament formation. Figure 4C compares a series of CAFM images focusing on a localized region corresponding to the square markers in Fig. 4 (A and B), showing the dynamic process of forming and rupturing a conducting filament within the device, offering an unprecedented real-time visualization of this resistive switching mechanism. Initially, the device exhibits an HRS with no detectable current at −3 V. As the bias is increased to −6 V, a high current region appears, suggesting the formation of a conducting filament. Immediately after the forming process, the application of −3 V yields a pronounced high-current area. This region, absent in the preforming state (first image), confirms the creation of a conducting filament, which then gradually decreases in intensity after 30 and 60 min, revealing the filament’s recoverable nature in contrast to a permanent dielectric breakdown. The current value measured from CAFM is not comparable to probe station measurements (which yield the results in Figs. 1 and 2) due to large contact resistance of the CAFM tip. Therefore, a larger forming voltage of 6 V was required during CAFM compared to the lower 4 V in Fig. 1A. Similarly, the almost complete resistance recovery in Fig. 4C does not mean that device retention is limited to 60 min, and the actual device retention is measured in Fig. 2A. A schematic illustration (Fig. 4D) shows the location of the conducting filaments in a cross-sectional view.

## DISCUSSION

In the following, we will provide a discussion of the guided filament formation and switching mechanism, and the potential limitations of the devices. The schematic in Fig. 4D illustrates the observed phenomenon where the filaments form preferentially at the vertical interfaces between the dissimilar composition grains in the nanocomposite switching layers, embodying a system that inherently guides filament formation. This guided filament formation can be attributed mainly to the fact that the interfaces between the WO_3_ and Ce_2_O_3_ grains act as vertical channels for the fast, preferential diffusion of oxygen vacancies because they are less ordered nanoscale regions in the nanostructure compared to the bulk grains (*53*). Such diffusion is also reversible for the rupture of the filaments, providing robust, stable resistive switching. The filaments are partial, connecting the top electrode and the epitaxial WO_3_ layer, as we observed from the STEM-EDS in Fig. 4 (C and D) that the WO_3_:Ce_2_O_3_ interfaces terminate at the thin epitaxial WO_3_. This model is suggested by the current-area relation in Fig. 2D—the “set current” (i.e., the current at +4 V during the transition from HRS to LRS) scales with the electrode area, whereas the “reset current” (current at −4 V during the transition from LRS to HRS) shows no such area dependence. In the set process, a partial filament (which does not extend continuously through the entire film) connects the top electrode to the epitaxial WO_3_:substrate interface. The electrons pass through the bottom electrode, overcome the energy barrier by the epitaxial interface layer and then pass through the conducting partial filament to the top electrode. The dominant impediment of a current is the Schottky-like barrier at the epitaxial interface, which scales with electrode size. Such an observation agrees with the finding of a Schottky emission model fitting in the set process of *I*-*V* curves, shown in fig. S12. In the reset process, the current is dominated by the ruptured filament. The conductive filament undergoes reoxidation, occurring predominantly at the vertical phase boundaries due to the fast diffusion path. The diffusion of oxygen back into the filament (or equivalently, the drift of oxygen vacancies out of the filament) reoxidizes and ruptures the conductive path. The reoxidation of oxygen vacancy-based conductive filaments is a phenomenon commonly observed in binary metal-oxide resistive switching devices (*54*). This process is inherently localized and thus does not scale with the electrode area. Increasing the device area does not lead to a proportional increase in current as the current is not determined by the WO_3_:substrate interface rather by the filaments. Our devices incorporate elements of both filamentary and interfacial switching. The scaling LRS resistance with area was also observed in another resistive switching system with both filamentary and interfacial conductions (*55*). In our nanocomposite, both filamentary and interfacial switching modes coexist: Predefined, partial filaments form preferentially along the WO_3_:Ce_2_O_3_ phase boundaries, whereas the epitaxial WO_3_:substrate interface contributes an additional interfacial switching element. Together, these mechanisms underpin the robust and reproducible resistive switching behavior observed in our devices.

In extended endurance tests for $2\times 10^{6}$ cycles (fig. S13), the devices enter increasingly large fluctuations in both the LRS and HRS, although they recover a more stable switching performance beyond 1.5 × 10^6^ cycles. This intermediate instability of the two states likely originates from more nonrecoverable oxidation and reduction of the filaments near the interface with continued switching, a phenomenon commonly observed in filamentary binary oxide systems (*54*). We also notice a progressive drift of the LRS toward higher resistance, thereby narrowing the memory window; this could be attributed to the depletion or reduced mobility of oxygen vacancies upon repeated cycling, a behavior reported in other WO*_x_*-based devices (*56*). By confining the conduction pathways within predefined interfaces, our nanocomposite design reduces filamentary stochasticity and delays filament breakdown, offering improved performance compared with other filamentary WO_3_. Further improvements may involve using electrodes that serve as oxygen vacancy reservoirs or applying doping strategies to stabilize vacancy concentrations to increase oxygen vacancy mobility and delay the depletion of oxygen vacancies. These modifications could potentially extend the endurance beyond the 10^6^ cycles achieved here.

The CAFM current map in Fig. 4B shows that the width of the filaments ranges from 20 to 40 nm, where it is worth mentioning that this measured size represents an upper limit due to inherent limitations in the spatial resolution of the measurement. The finite size of the CAFM tip (≈25 nm) and the possible diffusion of charge carriers during current measurement contribute to an overestimation of the actual filament width, and quantifying the latter effect is challenging. Supporting this, high-resolution STEM images (Fig. 4A) reveal that the width of the vertical interfaces, which represent the sites of filament formation, is ~5 to 10 nm, and the actual conducting filaments are confined within these narrow vertical regions. Therefore, although the CAFM provides valuable information about the location of the filaments, the true filament widths are likely closer to 5 to 10 nm. This finer scale of filament formation aligns with the nanostructure of the vertical interfaces and underscores the precision of the nanocomposite’s design in guiding filament sites. However, considering the AFM topography in Fig. 4A, we note that the grain sizes for WO_3_ are around 80 to 100 nm, which could set a lower limit for scaling down of the devices. Nonetheless, the size of the WO_3_ grains and Ce_2_O_3_ grains in the nanocomposite could be reduced by increasing the laser frequency during the deposition process, providing the potential for further miniaturization of the memory devices (*57*). However, we observed that an additional 6 V of forming voltage was required for devices with 25-μm electrode diameters (see fig. S14) to establish the same *I*-*V* curve as for larger devices. We attribute this difference to the relatively lower number of phase boundaries accessible within the coverage area of a smaller electrode, which leads to potentially less well-connected phase boundaries with larger variation, hence requiring a larger voltage to form partial guided filaments along “optimal” boundaries. In future work, we aim to refine the distribution and structure of the WO_3_:Ce_2_O_3_ interfaces to enhance device performance at smaller lateral dimensions. Our present focus is to demonstrate the concept of guided filament endurance improvement, with better scalability serving as a subsequent optimization goal.

Although Pt top electrodes may limit industrial compatibility, additional measurements showing excellent performance were done on devices with W (tungsten) top electrodes (see fig. S15). W and Pt have distinctly different work functions [≈5.8 eV for Pt and ≈4.7 eV for W (*58*)], suggesting that the resistive switching originates in the nanocomposite regardless of the top electrode, aiding future industrial compatibility. As for the bottom electrode, we used a single-crystal, semiconducting Nb:SrTiO_3_ substrate as it provides an atomically flat surface, minimal native oxide layer, and strong structural compatibility for epitaxial WO_3_. These features allowed us to investigate the guided filament approach in a standard reference system. An earlier work suggests that the resistance contribution from Nb:SrTiO_3_ is negligible compared with the total device resistance, and reference devices with Pt electrodes deposited directly onto Nb:SrTiO_3_ showed much lower endurance and retention (*7*). Our thin film design strategy for the WO_3_-based switching layers results in a resistive switching model that takes the advantages of the high retention from the filamentary switching mode with sufficiently high on/off ratios compared with other WO_3_-based memristive devices and enhances it with higher endurance and uniformity due to the robust predefined filaments, which form along the vertical interfaces between WO_3_ and Ce_2_O_3_ grains.

In summary, excellent resistive switching behavior was demonstrated in the memristive devices fabricated using WO_3_:Ce_2_O_3_ nanocomposite films. The devices show endurance performance with a stable on/off ratio ≳10 up to 10^6^ switching cycles and good device-to-device uniformity. Binary state retention was measured for 10^5^ s, together with the feasibility of attaining multistate retention. The unique structure of the nanocomposite reveals a clear phase separation between WO_3_ and Ce_2_O_3_ grains. The observation of conducting filaments by CAFM highlights a guided filament model, where filaments formed preferentially at the vertical interfaces in the nanocomposites. The devices’ nonvolatility and long-term functionality were achieved by carefully engineering the two-phase nanocomposite. Overall, our comprehensive study demonstrates a proof-of-concept nanocomposite system that enables advanced resistive switching behavior for memory applications. The excellence of endurance and retention, combined with the observed guided filament mechanism, underscores the potential of a careful materials design approach for high-performance devices suitable for integration into high-performance nonvolatile memories and neuromorphic computing systems.

## MATERIALS AND METHODS

### PLD of films

For nanocomposite films, the PLD target was prepared by WO_3_ and CeO_2_ powders of purity >99.9%. The two materials in powders were weighed in a 1:1 cation stoichiometric ratio, manually ground for 60 min, pressed into a pellet, and sintered at 1100°C for 8 hours. Except for the temperature, which was varied as described in the main text, the following parameters were used for all depositions: laser fluence, 1.5 J/cm^2^; deposition time, 50 min; laser frequency, 3 Hz; and oxygen pressure, 100 mtorr. After the deposition, the films were cooled down to room temperature at 10°C/min in vacuum. Reference WO_3_ thin films were deposited from a pure WO_3_ PLD target prepared using WO_3_ powders of purity >99.9% pressed into a pellet and sintered at 1200°C for 6 hours. All other deposition parameters are identical to the nanocomposite films.

### XRD measurements

XRD measurements were carried out using a Panalytical Empyrean Diffractometer system with parallel beam optics, Cu Kα1 radiation, a single-point proportional detector, or a PIXcel3D position-sensitive detector. Rietveld refinement of the 2θ-ω diffraction pattern was performed with TOPAS Academic V7 software (*59*).

### Sample fabrication

All films were deposited directly by PLD onto the 5 mm–by–5 mm (001)-oriented single-crystal 0.5 wt % Nb-doped SrTiO_3_ (Nb:SrTiO_3_) substrates, serving as the bottom electrode. The films were then annealed at 350°C for 3 hours in an oxygen-rich atmosphere in a tube furnace, with intake oxygen flow rate of 20 SCCM (standard cubic centimeter per minute). Top electrodes (Pt and W) were sputter deposited following a standard lift-off process. An AZ 4533 UV photoresist was spin coated (8000 rpm for 30 s, acceleration time of 10 s, deceleration time of 5 s) on the sample surface, soft baked at ≈100°C for 2 min, and exposed to ultraviolet (UV) light for 10 s through a mask. The resist was developed with the AZ 351 B developer, and the top metal was sputtered on them. The resist lift-off was carried out by submerging the samples in acetone for about 5 min and carefully sonicating the containing beaker to remove the unexposed UV resist and lift off the metal on top.

### Electrical measurements

Electrical measurements were carried out with a computer-controlled Keysight B2912A, connected to an EverBeing manual probe station with triax cables (*60*). The probe material was tungsten, and the bottom contact was established by conductive silver paint in contact with the Nb:SrTiO_3_. Endurance measurements were programmed by pulses: Each complete cycle involved four sequential pulses, including (1) a 4-V set pulse for 4 ms, (2) a 0.1-V read pulse for 4 ms, (3) a −4-V reset pulse for 4 ms, and (4) a 0.1-V read pulse for 4 ms, repeated continuously up to 10^6^ cycles. The rise/fall times of these pulses are not well controlled but should be on the order of 50 μs as per the instrument manual. Retention measurements were programmed as write pulses of ±4 V for 1 s, followed by read pulses of 0.1 V for 4 ms, repeated every 1 s for the first 12 hours (43,200 s), and then the device was monitored for the first 1000 s of the 13th, 14th, 15th, 16th, 17th, 19th, 21st, 23rd, 25th, and 28th hour, giving total of 101,800 s.

### Atomic force microscopy

The surface topography of the films was characterized using a Bruker Dimension atomic force microscope and a Tap300Al-G tip (40 N/m, 300 kHz, BudgetSensors) in the peak force tapping mode. Current measurements were carried out using a Bruker Dimension Icon Pro and a PtIr-coated Sb-doped Si SCM PIC tip in tunneling AFM (TUNA) mode. The bottom of the sample was electrically connected to the AFM tool by gluing it to a metal disc with conductive Ag paint. Voltages were applied to the bottom of the sample while the CAFM tip was grounded.

### Scanning transmission electron microscopy–energy-dispersive X-ray spectroscopy

All cross-sectional STEM-EDS samples were prepared using either focused ion beam in an FEI Helios Nanolab SEM/FIB or diamond lapping the film for mechanical polishing followed by mechanical grinding until the desired thickness was achieved. Once the samples reached the appropriate thickness, an argon ion milling system (Gatan Precision) was used for the final polishing. All images were captured using either a Thermo Fisher Scientific Spectra 300 operating at 300 kV or a Thermo Fisher Scientific TALOS 200X TEM operated at 200 kV. Image analysis was performed by open-source Hyperspy Python packages (*61*).

### X-ray photoelectron spectroscopy

XPS was performed by Thermo Fisher Scientific Escalab 250Xi instrument with monochromatic Al Kα radiation (*h*ν = 1486.6 eV) at ≈1 × 10^−9^ torr. The spectra were acquired after gentle sputter cleaning the sample surfaces by 200-eV Ar ions for 4000 s to remove native surface oxides. Using this ultralow Ar ion energy range avoids damaging core levels and thus inducing artifacts.

## Supplementary Materials

### This PDF file includes:

Figs. S1 to S15 References
