# Supplementary material for: Engineered high endurance in WO3-based resistive switching devices via a guided filament approach
Source: Sci Adv. 2025 May 16;11(20):eadt9789. doi: 10.1126/sciadv.adt9789 (PMC12083524; doi:10.1126/sciadv.adt9789)
Supplement: Supplementary file 1 — Figs. S1 to S15 References [file sciadv.adt9789_sm.pdf]

Science Advances
AAAS

Supplementary Materials for

Engineered high endurance in WO_3-based resistive switching devices via a guided filament approach

Ziyi Yuan *et al.*

Corresponding author: Ziyi Yuan, zy341@cam.ac.uk; Judith L. MacManus-Driscoll, jld35@cam.ac.uk

Sci. Adv. **11**, eadt9789 (2025)
DOI: 10.1126/sciadv.adt9789

This PDF file includes:

Figs. S1 to S15
References

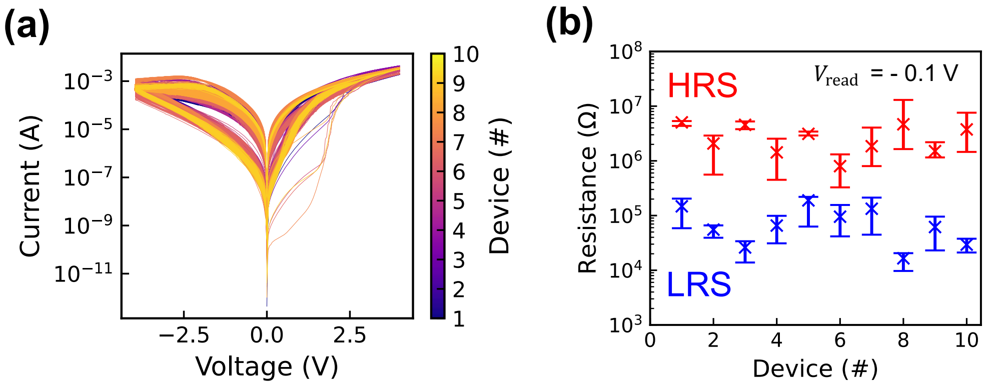

Fig. S1. Uniform DC switching performance across multiple devices.
(a) 100-cycle DC switching measurements on 10 devices with different colors indicating different devices. The overlapping 100-cycle DC switching curves from 10 devices suggest high uniformity of the I–V profiles across multiple devices. **(b)** The average high resistance state (HRS) and low resistance state (LRS) values of each device in (a) extracted from the 100-cycle curves at -0.1 V. Error bars indicate the minimum and maximum resistance among the 100 cycles in each device. Similar to the pulsed measurement in **Fig. 1D** in the main text, there is not a single device showing a LRS higher than the lowest possible HRS value among all devices, and vice versa. These data suggest consistent device-to-device performance in DC switching.

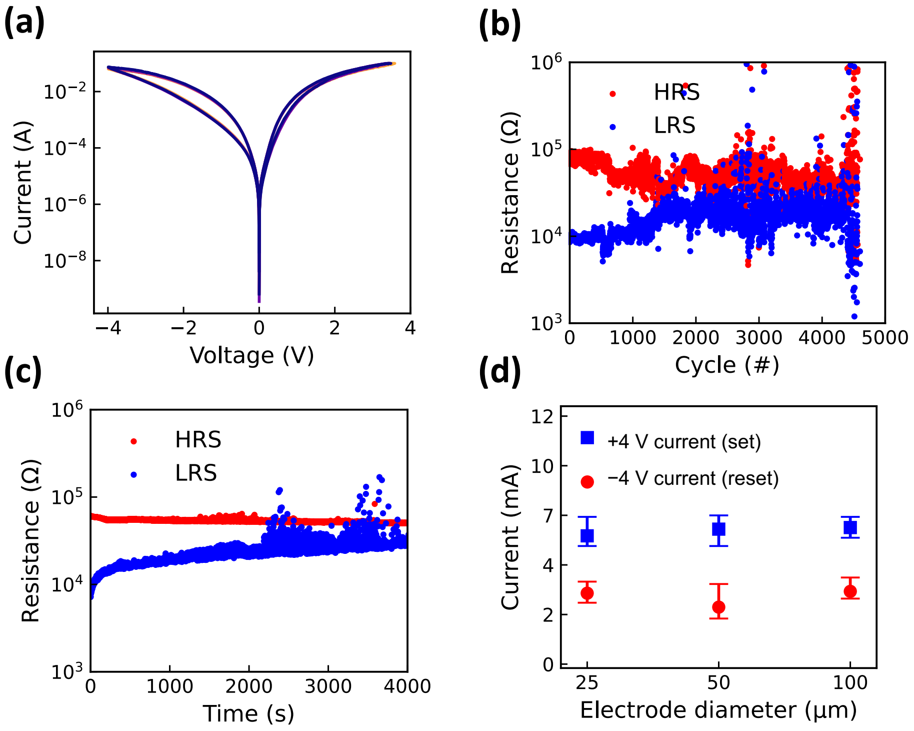

Fig. S2. I–V, endurance, and retention measurements on devices based on pure WO_3 films grown on (001)-oriented 0.5 wt% Nb-doped $SrTiO_3$ under the same conditions as the nanocomposite films.

(a) Initial five I–V curves. **(b)** Endurance measurements of 5000 switching cycles on the device in (a). The HRS and LRS collapse at about 1000 cycles. The set and reset voltages were ±4 V; the read voltage was 0.1 V. **(c)** Two-state retention measurements on the device in (a). The set, reset and read voltages were the same as in (b). **(d)** Dependence of the currents at +4 V and -4 V in the I–V curves on the electrode size. Five devices, each with top electrode diameters of 25, 50, and 100 µm, were tested for 5 cycles. Error bars include the maximum and minimum current of the 5 cycles from each of the 5 devices. No area dependence is observed, indicating a clear filamentary switching.

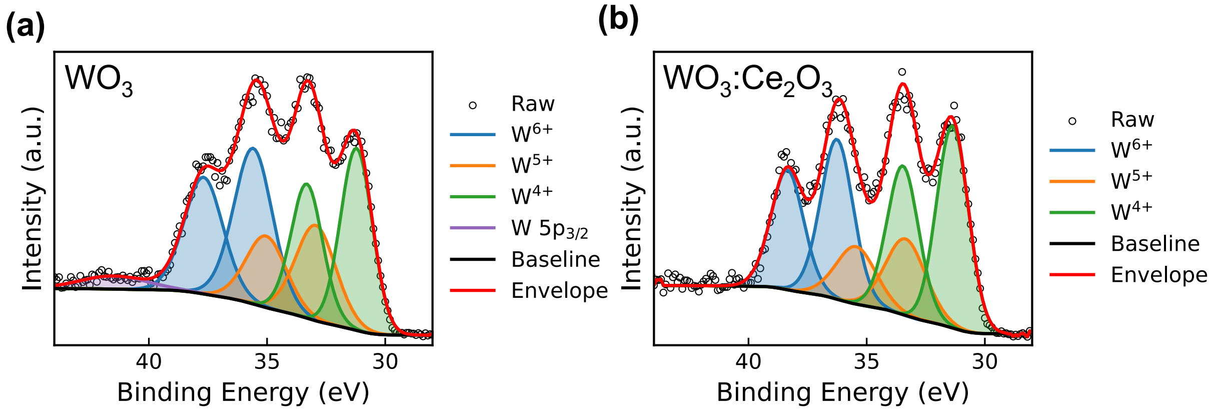

Fig. S3. X-ray photoelectron spectroscopy (XPS) spectra of W 4f core level.
(a) The reference pure WO_3 and **(b)** the nanocomposite film fabricated under identical conditions. After calibrating the binding energy scales against Fermi edges, we observed an approximately 0.9 eV peak shift in the nanocomposite W 4f spectrum compared with that of the reference WO_3 sample. This indicates a higher concentration of oxygen vacancies in the WO_3:Ce_2O_3 nanocomposite thin films than in pure WO_3, which can largely contribute to more gradual and better switching performance of the composite devices. Both spectra consist of convoluted peaks. The W 4f core level spectra were deconvoluted after the Shirley-type background subtraction by maintaining the same $4f_{5/2}$–$4f_{7/2}$ binding-energy separations (2.1 eV), line shapes (Gaussian–Lorentzian), full-width-at-half-maximum values (1.6 eV), and $4f_{5/2}$:$4f_{7/2}$ area ratios (3:4), while peak areas and positions were changed. The W 4f spectra of both samples consist of three doublets corresponding to 6+, 5+, and 4+ oxidation states. The presence of lower oxidation states (W^{5+} and W^{4+}) was already reported for PLD-grown WO_3 thin films (*62*). While the W^{6+}, W^{5+} and W^{4+} concentrations in the WO_3 sample are 37.8%, 23.9%, and 35.3%, respectively, (and 3% of W 5p3/2), the nanocomposite is composed of 35.7% W^{6+}, 21.0% W^{5+}, and 43.3% W^{4+}. This clearly shows an increase in the W^{4+} in the WO_3:Ce_2O_3, indicating a higher oxygen vacancy concentration in the WO_3 phases of the nanocomposite film compared to the pure WO_3 film. However, XPS cannot reliably quantify the total oxygen vacancy concentration for the entire nanocomposite due to the presence of the additional CeO_x phase and the inherent limitations of the XPS measurement in the analysis of O 1s spectrum (*63*).

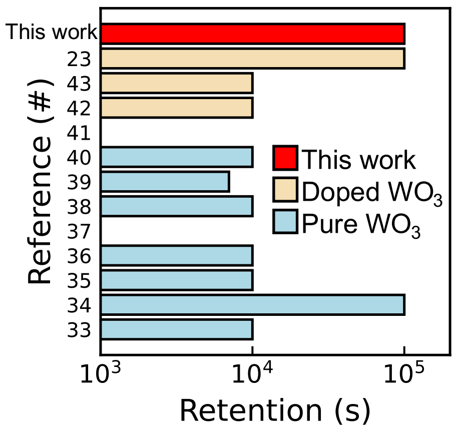

Fig. S4. Retention comparison of our work with the same WO_3 filamentary resistive switching devices as in the endurance benchmark in Fig. 1C in the main text.
Our nanocomposite device outperforms or meets all previous work. Two studies did not report retention data for their devices; therefore, the corresponding lines are left blank.

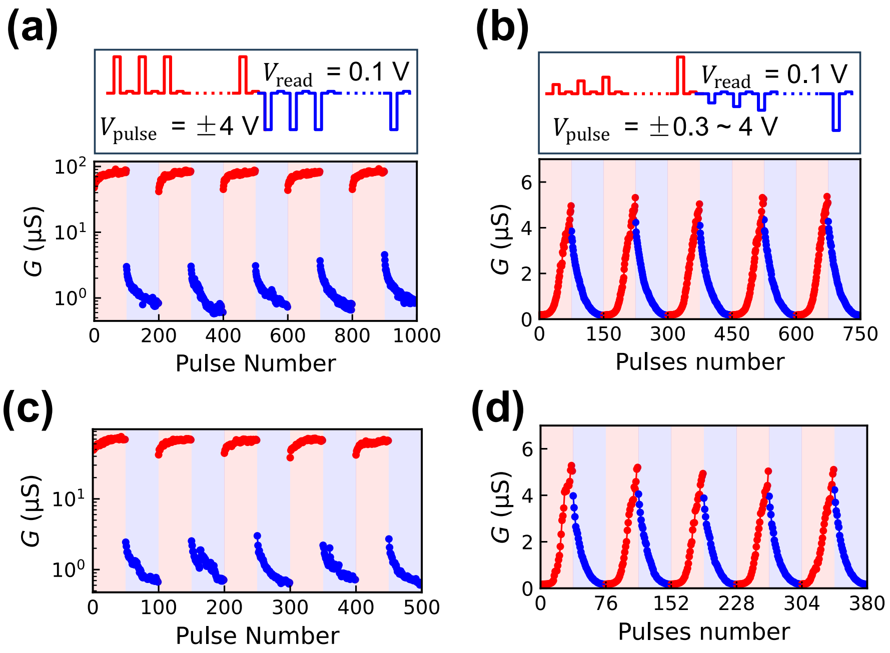

Fig. S5. Analog switching behavior of the nanocomposite devices for neuromorphic applications.

(a) Analog switching of the device by programming with fixed write voltage pulses of ±4 V for a total of 200 pulses per cycle of potentiation (red region) and depression (blue region). The pulse length is 100 ms. **(b)** Analog switching of the device by programming with incremental write voltage pulses from ±0.3 V to 4 V in steps of 0.05 V for a total of 70 pulses per cycle of potentiation (red region) and depression (blue region). The pulse length is 100 ms. **(c)** Device programming with fixed pulses as in (a), but with 100 pulses per cycle and a 5 ms pulse length. **(d)** Device programming with incremental pulses as in (b), but with 76 pulses per cycle and a 10 ms pulse length. The pulse lengths in (c) and (d) are comparable to those in the endurance measurements (4 ms). Read pulses are 0.1 V for all measurements.

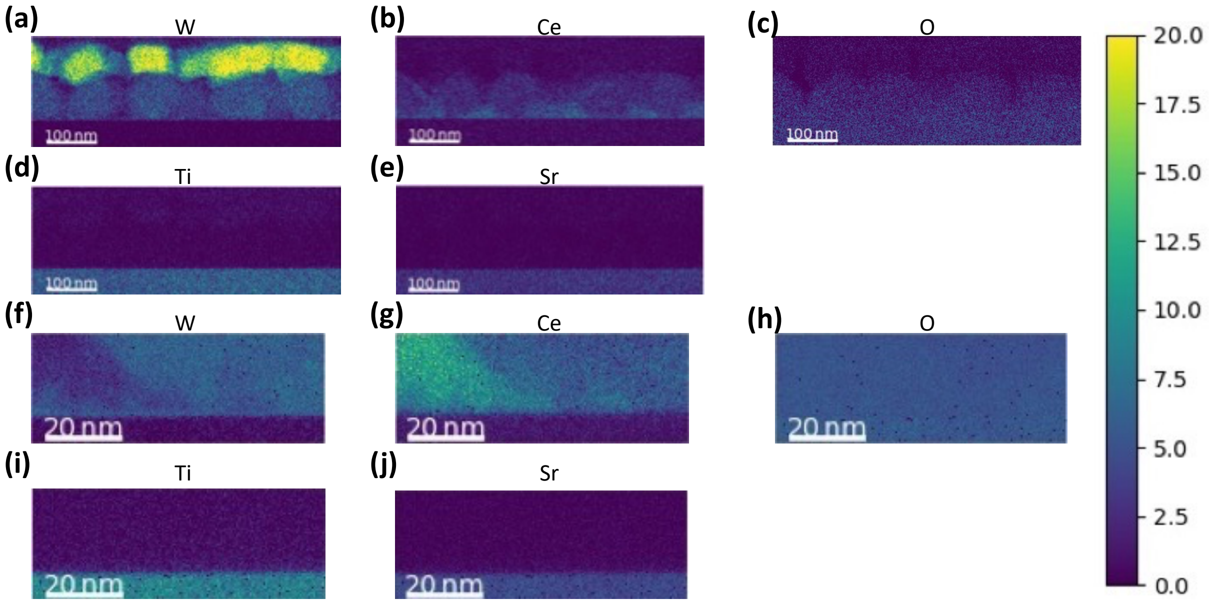

Fig. S6. Cross-sectional energy-dispersive X-ray spectroscopy (EDS) atomic percent maps of the major elements in the nanocomposite.

(a-e) Atomic percentage maps of W, Ce, O, Ti, and Sr corresponding to **Fig. 3B** in the main text. **(f-j)** Atomic percentage maps of W, Ce, O, Ti, and Sr corresponding to **Fig. 3D** in the main text. The high W signal in (a) originates from the W electrode on top of the film.

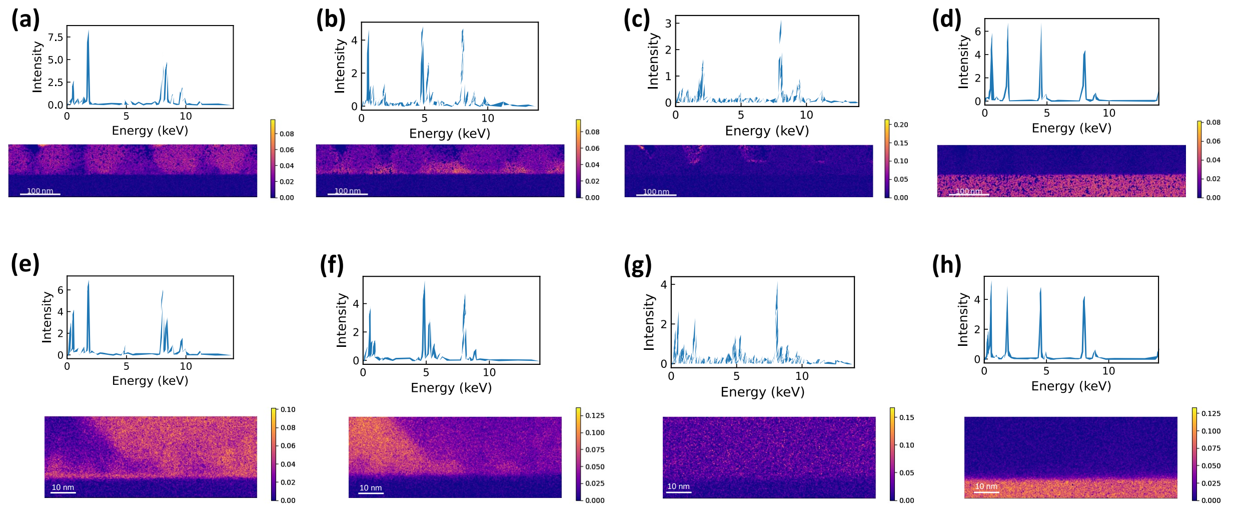

Fig. S7. Non-negative matrix factorization analysis details of the EDS spectra.

(a-d) Four factors showing different compositional structures corresponding to the EDS spectrum in **Fig. 3B** in the main text. The four structures are: W-rich phase, Ce-rich phase, Pt residual from lamella preparation, and substrate, respectively. **(e-h)** Four factors showing different compositional structures corresponding to the EDS spectrum in **Fig. 3D** in the main text. The four structures are: W-rich phase, Ce-rich phase, unknown (likely to be oxygen atoms), and substrate, respectively.

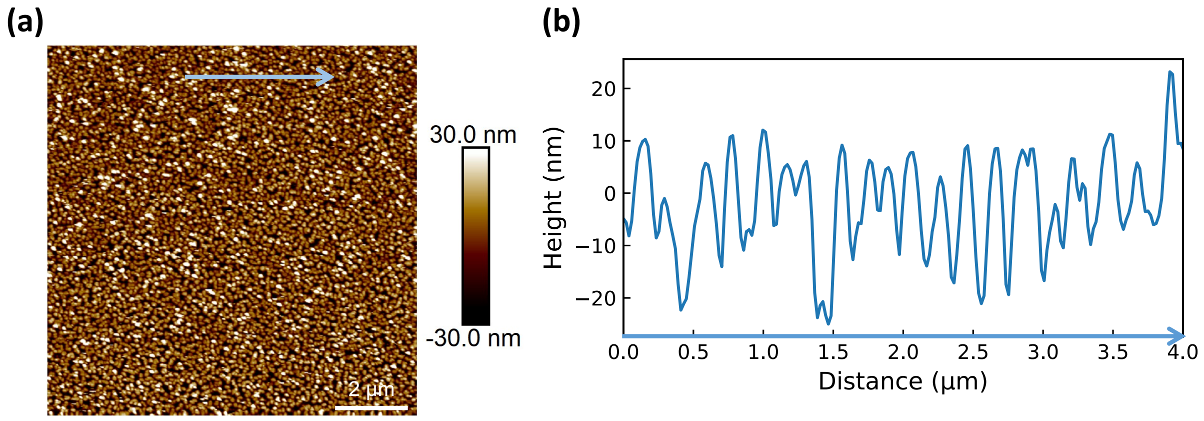

Fig. S8. AFM topography measurement on the film deposited at 900 °C.

(a) AFM topography showing circular grains uniformly distributed across a 10 µm by 10 µm area. **(b)** A line scan along an arbitrary direction. The dimensions and spacing of the grains (approximately 120 nm) align with the observed STEM patterns in **Fig. 3B** in the main text, indicating a consistent structure from the topography to the cross-sectional view.

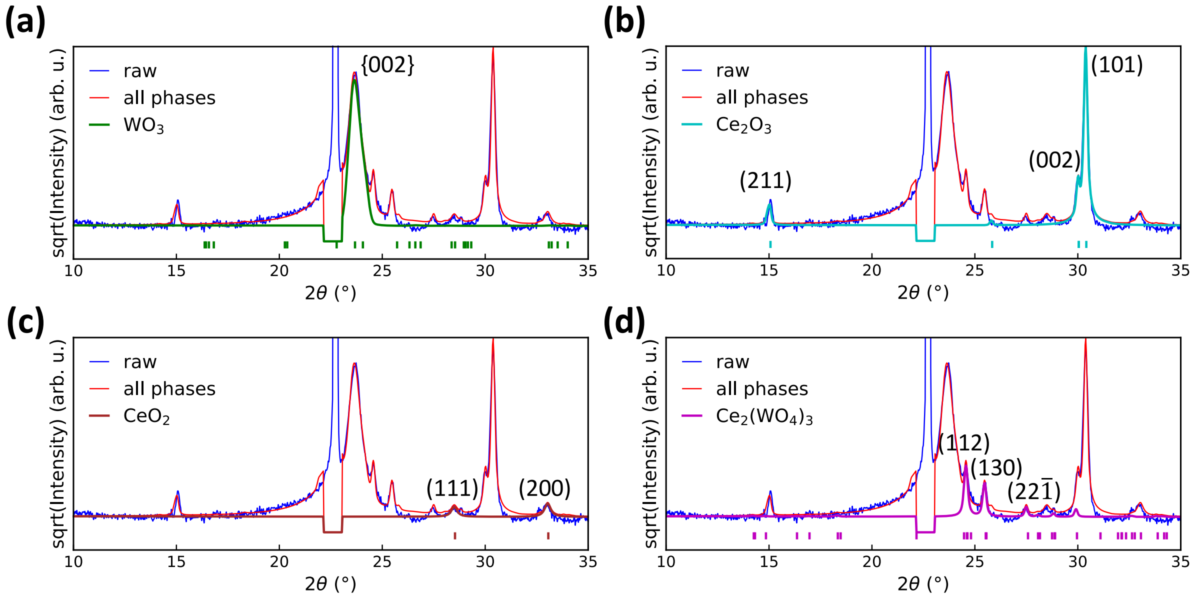

Fig. S9. Rietveld refinement of the 2*θ*-*ω* diffraction pattern in Fig. 3E in the main text.
(a) WO_3. **(b)** Ce_2O_3. **(c)** CeO_2. **(d)** $Ce_2(WO_4)_3$. Crystal structures were retrieved from the Inorganic Crystal Structure Database, ICSD (WO_3 (*64*), Ce_2O_3 (*65*), $Ce_2(WO_4)_3$ (*66*), and CeO_2 (*67*)). A Chebyshev function with two parameters was used to model the background. The March–Dollase model for preferred orientation was applied on the following planes: (010) and (110) for WO_3; (001) for CeO_2; (001) for Ce_2O_3; (130) and (112) for $Ce_2(WO_4)_3$. In addition to the main peaks of WO_3 and Ce_2O_3, there are minor peaks due to CeO_2 and $Ce_2(WO_4)_3$.

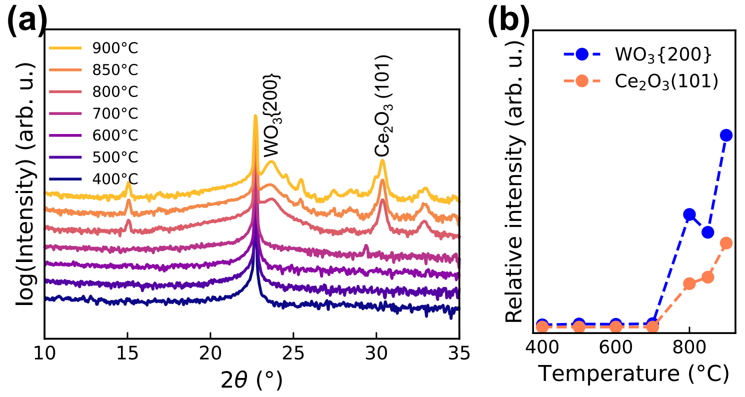

Fig. S10. 2*θ*-*ω* X-ray diffraction of the nanocomposite films deposited at various temperatures.

(a) 2*θ*-*ω* scans of the nanocomposite films on $SrTiO_3$ (STO) substrates with varying deposition temperatures. The growth of nanocomposites only occurred at or above 800 °C. **(b)** Temperature dependence of the peak intensities of WO_3 {200} and Ce_2O_3 (101), scaled to the intensity of the STO (001) peak in the respective scans from (a), describing the influence of deposition temperature on the crystalline growth of our films. The intensity of the WO_3 {200} peak and the Ce_2O_3 (101) peak increases with increasing deposition temperature, suggesting a threshold between 700 °C and 800 °C conducive to optimal crystalline growth.

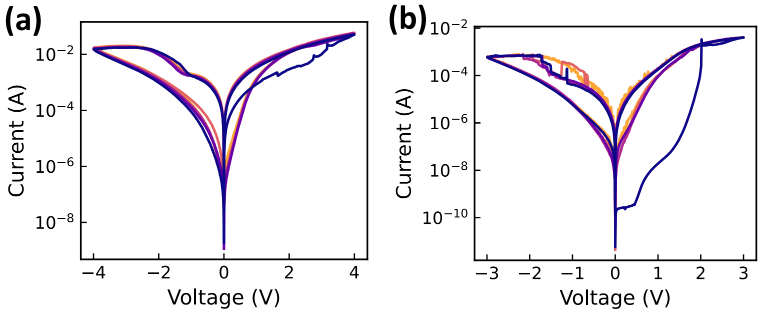

Fig. S11. I–V measurements on devices on the nanocomposite films deposited at 800 °C and 850 °C.

(a) Initial five I–V curves of a device based on the film deposited at 800 °C. **(b)** Initial five I–V curves of a device based on the film deposited at 850 °C. These I–V curves are less stable compared to those from films deposited at 900 °C in **Fig. 1A** in the main text.

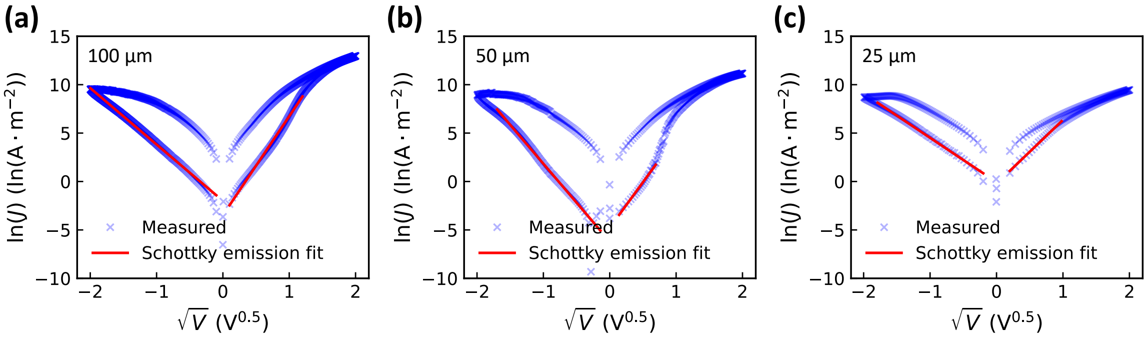

Fig. S12. Schottky emission electronic transport model (*68*) fitted to the measured I-V curves in Fig. 2C in the main text, $\ln(J)$ vs. $\sqrt{V}$, with all $R^2 > 0.98$.

(a) Fitting for the 100 µm electrode device. **(b)** Fitting for the 50 µm electrode device. **(c)** Fitting for the 25 µm electrode device. A linear fitting in the set process suggests the contribution of Schottky emission to the electronic transport processes within the device.

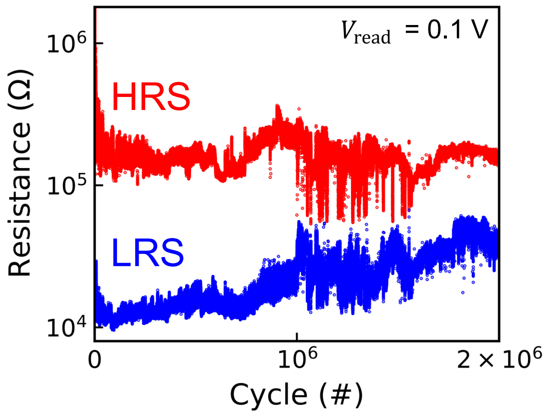

Fig. S13. Extended endurance test for 2×10^6 cycles revealing the effective failure of the device after 10^6 cycles.

Even though the device recovers beyond about 1.5×10^6 cycles, we do not claim more than 10^6 cycles of endurance, as the memory window is effectively closed in the intermediate failure sequence. Both the LRS and HRS fluctuate significantly, and LRS is slowly drifting towards higher resistance, culminating in state collapse.

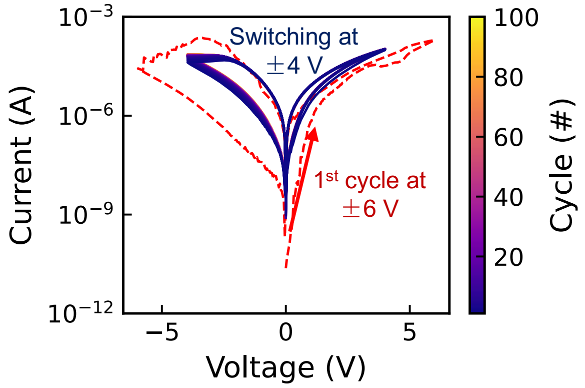

Fig. S14. Initial 100 I–V curves of the 25 µm top electrode device presented in Fig. 2C in the main text.

The device required 6 V to form, indicated by the red dashed line in the 1st cycle, and then ± 4 V was used for the subsequent 100 cycles. This behavior is different in devices with 50 µm and 100 µm electrodes where forming did not require higher voltage than switching.

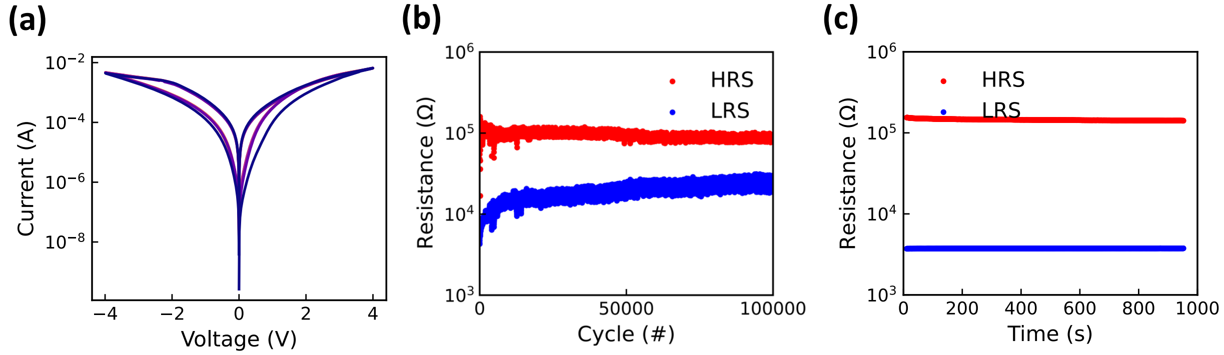

Fig. S15. I–V, endurance, retention measurements on devices with W top electrodes on the optimum (deposited at 900 °C) nanocomposite film.

(a) Initial five I–V curves. **(b)** Endurance measurement of 10^5 switching cycles on the device in (a). Set and reset voltages were ±4 V; the read voltage was 0.1 V. **(c)** Two-state retention measurements on the device in (a). The set, reset, and read voltages were the same as in (b).

REFERENCES AND NOTES

1. V. Castro, M. Georgiou, T. Jackson, I. R. Hodgkinson, L. Jackson, S. Lockwood, Digital data demand and renewable energy limits: Forecasting the impacts on global electricity supply and sustainability. *Energy Policy* **195**, 114404 (2024).

2. S. Yu, Neuro-inspired computing with emerging nonvolatile memorys. *Proc. IEEE* **106**, 260–285 (2018).

3. K. Krishnan, S. Mohammad Tauquir, S. Vijayaraghavan, R. Mohan, Configurable switching behavior in polymer-based resistive memories by adopting unique electrode/electrolyte arrangement. *RSC Adv.* **11**, 23400–23408 (2021).

4. D. Kumar, R. Aluguri, U. Chand, T. Y. Tseng, Metal oxide resistive switching memory: Materials, properties and switching mechanisms. *Ceram. Int.* **43**, S547–S556 (2017).

5. E. Gale, TiO_2-based memristors and ReRAM: Materials, mechanisms and models (a review). *Semicond. Sci. Technol.* **29**, 104004 (2014).

6. S. Brivio, S. Spiga, D. Ielmini, HfO_2-based resistive switching memory devices for neuromorphic computing. *Neuromorph. Comput. Eng.* **2**, 042001 (2022).

7. M. Hellenbrand, B. Bakhit, H. Dou, M. Xiao, M. O. Hill, Z. Sun, A. Mehonic, A. Chen, Q. Jia, H. Wang, J. L. MacManus-Driscoll, Thin-film design of amorphous hafnium oxide nanocomposites enabling strong interfacial resistive switching uniformity. *Sci. Adv.* **9**, eadg1946 (2023).

8. J. J. Yang, M.-X. Zhang, J. P. Strachan, F. Miao, M. D. Pickett, R. D. Kelley, G. Medeiros-Ribeiro, R. S. Williams, High switching endurance in TaO_x memristive devices. *Appl. Phys. Lett.* **97**, 232102 (2010).

9. B. W. Fowler, Y.-F. Chang, F. Zhou, Y. Wang, P.-Y. Chen, F. Xue, Y.-T. Chen, B. Bringhurst, S. Pozder, J. C. Lee, Electroforming and resistive switching in silicon dioxide resistive memory devices. *RSC Adv.* **5**, 21215–21236 (2015).

10. B. Qu, A. Younis, D. Chu, Recent progress in tungsten oxides based memristors and their neuromorphological applications. *Electron. Mater. Lett.* **12**, 715–731 (2016).

11. S. Lamichhane, S. Sharma, M. Tomar, V. Gupta, Non-volatile resistive switching in WO_3 thin films. *AIP Conf. Proc.* **2220**, 040035 (2020).

12. M.-J. Lee, C. B. Lee, D. Lee, S. R. Lee, M. Chang, J. H. Hur, Y.-B. Kim, C.-J. Kim, D. H. Seo, S. Seo, U.-I. Chung, I.-K. Yoo, K. Kim, A fast, high-endurance and scalable non-volatile memory device made from asymmetric Ta_2O_{5-x}/TaO_{2-x} bilayer structures. *Nat. Mater.* **10**, 625–630 (2011).

13. R. Sohal, C. Walczyk, P. Zaumseil, D. Wolansky, A. Fox, B. Tillack, H.-J. Müssig, T. Schroeder, Thermal oxidation of chemical vapour deposited tungsten layers on silicon substrates for embedded non-volatile memory application. *Thin Solid Films* **517**, 4534–4539 (2009).

14. U.S. Geological Survey, Mineral commodity summaries 2024 (U.S. Geological Survey, 2024), 212 pp.

15. W. C. Chien, Y. C. Chen, E. K. Lai, F. M. Lee, Y. Y. Lin, A. T. H. Chuang, K. P. Chang, Y. D. Yao, T. H. Chou, H. M. Lin, M. H. Lee, Y. H. Shih, K. Y. Hsieh, C.-Y. Lu, A study of the switching mechanism and electrode material of fully CMOS compatible tungsten oxide ReRAM. *Appl. Phys. A* **102**, 901–907 (2011).

16. Z.-H. Huang, H. Li, W.-H. Li, G. Henkelman, B. Jia, T. Ma, Electrical and structural dual function of oxygen vacancies for promoting electrochemical capacitance in tungsten oxide. *Small* **16**, e2004709 (2020).

17. A. Sawa, Resistive switching in transition metal oxides. *Mater. Today* **11**, 28–36 (2008).

18. S. Munjal, N. Khare, Advances in resistive switching based memory devices. *J. Phys. D Appl. Phys.* **52**, 433002 (2019).

19. M. Asif, A. Kumar, Resistive switching in emerging materials and their characteristics for neuromorphic computing. *Mater. Today Electron.* **1**, 100004 (2022).

20. K. Rudrapal, A. Mukherjee, V. Adyam, A. Roy Chaudhuri, Modulation of resistive switching properties of non-stoichiometric WO_{3-x} based asymmetric MIM structure by interface barrier modification. *J. Appl. Phys.* **129**, 235302 (2021).

21. W.-J. Chen, C.-H. Cheng, P.-E. Lin, Y.-T. Tseng, T.-C. Chang, J.-S. Chen, Analog resistive switching and synaptic functions in WO_x/TaO_x bilayer through redox-induced trap-controlled conduction. *ACS Appl. Electron. Mater.* **1**, 2422–2430 (2019).

22. Y. Bai, Y. Zhang, H. Wu, H. Qian, “High-density WO_x-based RRAM with a W-doped AlO_x insertion layer,” in *2013 5th IEEE International Memory Workshop* (IEEE, 2013), pp. 120–123.

23. Y.-E. Syu, T.-C. Chang, T.-M. Tsai, G.-W. Chang, K.-C. Chang, Y.-H. Tai, M.-J. Tsai, Y.-L. Wang, S. M. Sze, Silicon introduced effect on resistive switching characteristics of WO_X thin films. *Appl. Phys. Lett.* **100**, 022904 (2012).

24. M. Lanza, K. Zhang, M. Porti, M. Nafría, Z. Y. Shen, L. F. Liu, J. F. Kang, D. Gilmer, G. Bersuker, Grain boundaries as preferential sites for resistive switching in the HfO_2 resistive random access memory structures. *Appl. Phys. Lett.* **100**, 123508 (2012).

25. S. Tappertzhofen, S. Hofmann, Embedded nanoparticle dynamics and their influence on switching behaviour of resistive memory devices. *Nanoscale* **9**, 17494–17504 (2017).

26. S. Lee, A. Sangle, P. Lu, A. Chen, W. Zhang, J. S. Lee, H. Wang, Q. Jia, J. L. MacManus-Driscoll, Novel electroforming-free nanoscaffold memristor with very high uniformity, tunability, and density. *Adv. Mater.* **26**, 6284–6289 (2014).

27. G. U. Siddiqui, M. M. Rehman, K. H. Choi, Resistive switching phenomena induced by the heterostructure composite of $ZnSnO_3$ nanocubes interspersed ZnO nanowires. *J. Mater. Chem. C* **5**, 5528–5537 (2017).

28. Z. Guo, G. Liu, Y. Sun, Y. Zhang, J. Zhao, P. Liu, H. Wang, Z. Zhou, Z. Zhao, X. Jia, J. Sun, Y. Shao, X. Han, Z. Zhang, X. Yan, High-performance neuromorphic computing and logic operation based on a self-assembled vertically aligned nanocomposite $SrTiO_3$:MgO film memristor. *ACS Nano* **17**, 21518–21530 (2023).

29. H. Dou, X. Gao, D. Zhang, S. Dhole, Z. Qi, B. Yang, M. N. Hasan, J.-H. Seo, Q. Jia, M. Hellenbrand, J. L. MacManus-Driscoll, X. Zhang, H. Wang, Electroforming-free HfO_2:CeO_2 vertically aligned nanocomposite memristors with anisotropic dielectric response. *ACS Appl. Electron. Mater.* **3**, 5278–5286 (2021).

30. Z. Hu, H. Dou, Y. Zhang, J. Shen, L. Ahmad, S. Han, E. G. Hollander, J. Lu, Y. Zhang, Z. Shang, Y. Cao, J. Huang, H. Wang, Integration of CeO_2-based memristor with vertically aligned nanocomposite thin film: Enabling selective conductive filament formation for high-performance electronic synapses. *ACS Appl. Mater. Interfaces* **16**, 64951–64962 (2024).

31. C. Zhao, C. Z. Zhao, M. Werner, S. Taylor, P. Chalker, P. King, Grain size dependence of dielectric relaxation in cerium oxide as high-k layer. *Nanoscale Res. Lett.* **8**, 172 (2013).

32. Y. S. Chiu, J. T. Liao, Y. C. Lin, S. C. Liu, T. M. Lin, H. Iwai, K. Kakushima, E. Y. Chang, High-permitivity cerium oxide prepared by molecular beam deposition as gate dielectric and passivation layer and applied to AlGaN/GaN power high electron mobility transistor devices. *Jpn. J. Appl. Phys.* **55**, 051001 (2016).

33. L. Tao, Z. Kailiang, W. Fang, S. Kuo, S. Wenxiang, Z. Jinshi, Electrical properties and conduction mechanism of RRAM with Al/WO_X/Cu structure. *ECS Trans.* **52**, 1003–1008 (2013).

34. K. P. Biju, X. Liu, S. Kim, M. Siddik, J. Shin, J. Lee, H. Hwang, Bipolar resistance switching in the Pt/WOx/W nonvolatile memory devices. *Curr. Appl. Phys.* **11**, e62–e65 (2011).

35. Y. Li, S. Long, Q. Liu, Q. Wang, M. Zhang, H. Lv, L. Shao, Y. Wang, S. Zhang, Q. Zuo, S. Liu, M. Liu, Nonvolatile multilevel memory effect in Cu/WO_3/Pt device structures. *Phys. Status Solidi Rapid Res. Lett.* **4**, 124–126 (2010).

36. J. Kim, J. Park, S. Kim, Bipolar switching characteristics of transparent WOX-Based RRAM for synaptic application and neuromorphic engineering. *Materials* **15**, 7185 (2022).

37. J. Shim, J. Park, K. Kwon, K. S. Lee, D. I. Son, K. Yu, Tungsten oxide nonvolatile memory devices using photothermal in-situ oxidation method. *Mater. Lett.* **272**, 127805 (2020).

38. H. Hu, A. Scholz, C. Dolle, A. Zintler, A. Quintilla, Y. Liu, Y. Tang, B. Breitung, G. C. Marques, Y. M. Eggeler, J. Aghassi-Hagmann, Inkjet-printed tungsten oxide memristor displaying non-volatile memory and neuromorphic properties. *Adv. Funct. Mater.* **34**, 2302290 (2024).

39. K. Rudrapal, G. Bhattacharya, V. Adyam, A. Roy Chaudhuri, Forming-free, self-compliance, bipolar multi-level resistive switching in WO_{3-x} based MIM device. *Adv. Electron. Mater.* **8**, 2200250 (2022).

40. Y. Cho, J. Kim, M. Kang, S. Kim, Analog resistive switching and artificial synaptic behavior of ITO/WO_X/TaN memristors. *Materials* **16**, 1687 (2023).

41. J. Pyo, H. Ha, S. Kim, Enhanced short-term memory plasticity of WOx-based memristors by inserting AlO_x thin layer. *Materials* **15**, 9081 (2022).

42. S. M. Hong, H.-D. Kim, M. J. Yun, J. H. Park, D. S. Jeon, T. G. Kim, Improved resistive switching properties by nitrogen doping in tungsten oxide thin films. *Thin Solid Films* **583**, 81–85 (2015).

43. M. N. Kozicki, C. Gopalan, M. Balakrishnan, M. Mitkova, A low-power nonvolatile switching element based on copper-tungsten oxide solid electrolyte. *IEEE Trans. Nanotechnol.* **5**, 535–544 (2006).

44. W. C. Chien, Y. C. Chen, E. K. Lai, Y. D. Yao, P. Lin, S. F. Horng, J. Gong, T. H. Chou, H. M. Lin, M. N. Chang, Y. H. Shih, K. Y. Hsieh, R. Liu, C.-Y. Lu, Unipolar Switching Behaviors of RTO WO_X RRAM. *IEEE Electron Device Lett.* **31**, 126–128 (2010).

45. S. Dutta, S. Panchanan, J. H. Yoo, S. Kumar, H. C. Yoo, S. I. Seok, G. Dastgeer, D. H. Yoon, Synaptic behavior of iodine-enriched copper-based perovskite memristors developed through a sustainable solution approach. *Adv. Funct. Mater.* **34**, 2410810 (2024).

46. S. Ali, M. F. Khan, M. A. Ullah, M. W. Iqbal, Magnesium-doped ZnO thin film memristors for enhanced synaptic plasticity and resistive switching in neuromorphic computing. *J. Alloys Compd.* **1015**, 178800 (2025).

47. S. Yu, Y. Wu, R. Jeyasingh, D. Kuzum, H.-S. P. Wong, An electronic synapse device based on metal oxide resistive switching memory for neuromorphic computation. *IEEE Trans. Electron Devices* **58**, 2729–2737 (2011).

48. V. Pandey, Origin of the Curie–Von Schweidler law and the fractional capacitor from time-varying capacitance. *J. Power Sources* **532**, 231309 (2022).

49. V. P. Pauca, J. Piper, R. J. Plemmons, Nonnegative matrix factorization for spectral data analysis. *Linear Algebra Appl.* **416**, 29–47 (2006).

50. F. Zhang, Y. Zhang, L. Li, X. Mou, H. Peng, S. Shen, M. Wang, K. Xiao, S.-H. Ji, D. Yi, T. Nan, J. Tang, P. Yu, Nanoscale multistate resistive switching in WO_3 through scanning probe induced proton evolution. *Nat. Commun.* **14**, 3950 (2023).

51. S. Cho, C. Yun, S. Tappertzhofen, A. Kursumovic, S. Lee, P. Lu, Q. Jia, M. Fan, J. Jian, H. Wang, S. Hofmann, J. L. MacManus-Driscoll, Self-assembled oxide films with tailored nanoscale ionic and electronic channels for controlled resistive switching. *Nat. Commun.* **7**, 12373 (2016).

52. R. D. Shannon, Revised effective ionic radii and systematic studies of interatomic distances in halides and chalcogenides. *Acta Cryst. A* **32**, 751–767 (1976).

53. H. Dou, M. Hellenbrand, M. Xiao, Z. Hu, S. Kunwar, A. Chen, J. L. MacManus-Driscoll, Q. Jia, H. Wang, Engineering of grain boundaries in CeO_2 enabling tailorable resistive switching properties. *Adv. Electron. Mater.* **9**, 2201186 (2023).

54. H.-S. P. Wong, H.-Y. Lee, S. Yu, Y.-S. Chen, Y. Wu, P.-S. Chen, B. Lee, F. T. Chen, M.-J. Tsai, Metal–oxide RRAM. *Proc. IEEE* **100**, 1951–1970 (2012).

55. S. Roy, B. Chakrabarti, E. Bhattacharya, Coexistence of interfacial and filamentary resistance switching in Ti/SiO_x/Au resistive memory devices. *IEEE Trans. Electron Devices* **70**, 5421–5427 (2023).

56. D. S. Hong, Y. S. Chen, Y. Li, H. W. Yang, L. L. Wei, B. G. Shen, J. R. Sun, Evolution of conduction channel and its effect on resistance switching for Au-WO_{3-x}–Au devices. *Sci. Rep.* **4**, 4058 (2014).

57. A. Chen, Z. Bi, Q. Jia, J. L. MacManus-Driscoll, H. Wang, Microstructure, vertical strain control and tunable functionalities in self-assembled, vertically aligned nanocomposite thin films. *Acta Mater.* **61**, 2783–2792 (2013).

58. G. N. Derry, M. E. Kern, E. H. Worth, Recommended values of clean metal surface work functions. *J. Vac. Sci. Technol. A* **33**, 060801 (2015).

59. A. A. Coelho, *TOPAS and TOPAS-Academic*: An optimization program integrating computer algebra and crystallographic objects written in C++. *J. Appl. Cryst.* **51**, 210–218 (2018).

60. M. Hellenbrand, Keysight B2912 Memristor characterisation, Apollo–University of Cambridge Repository (2024); https://doi.org/10.17863/CAM.109318.

61. F. de la Peña, E. Prestat, V. T. Fauske, P. Burdet, J. Lähnemann, P. Jokubauskas, T. Furnival, C. Francis, M. Nord, T. Ostasevicius, K. E. MacArthur, D. N. Johnstone, M.

Sarahan, J. Taillon, T. Aarholt, pquinn-dls, V. Migunov, A. Eljarrat, J. Caron, T. Nemoto, T. Poon, S. Mazzucco, actions-user, N. Tappy, N. Cautaerts, S. Somnath, T. Slater, M. Walls, pietsjoh, H. Ramsden, hyperspy/hyperspy: v2.0.1, version v2.0.1, Zenodo (2024); https://doi.org/10.5281/zenodo.10709941.

62. D. I. Miakota, R. R. Unocic, F. Bertoldo, G. Ghimire, S. Engberg, D. Geohegan, K. S. Thygesen, S. Canulescu, A facile strategy for the growth of high-quality tungsten disulfide crystals mediated by oxygen-deficient oxide precursors. *Nanoscale* **14**, 9485–9497 (2022).

63. T. J. Frankcombe, Y. Liu, Interpretation of oxygen 1s X-ray photoelectron spectroscopy of ZnO. *Chem. Mater.* **35**, 5468–5474 (2023).

64. T. Vogt, P. M. Woodward, B. A. Hunter, The high-temperature phases of WO_3. *J. Solid State Chem.* **144**, 209–215 (1999).

65. H. Bärnighausen, G. Schiller, The crystal structure of A-Ce_2O_3. *J. Less-Common Met.* **110**, 385–390 (1985).

66. T. Gressling, H. Müller-Buschbaum, Zur Kristallstruktur von $Ce_2(WO_4)_3$/On the crystal structure of $Ce_2(WO_4)_3$. *Zeitschrift für Naturforschung B* **50**, 1513–1516 (1995).

67. M. Yashima, S. Kobayashi, T. Yasui, Crystal structure and the structural disorder of ceria from 40 to 1497 °C. *Solid State Ion.* **177**, 211–215 (2006).

68. R. T. Tung, The physics and chemistry of the Schottky barrier height. *Appl. Phys. Rev.* **1**, 011304 (2014).
